# Transfer Durability of Line-Patterned Replica Mold Made of High-Hardness UV-Curable Resin

**DOI:** 10.3390/nano10101956

**Published:** 2020-10-01

**Authors:** Tetsuma Marumo, Shin Hiwasa, Jun Taniguchi

**Affiliations:** 1 Department of Applied Electronics, Tokyo University of Science, 6-3-1, Niijyuku, Katsushika-ku, Tokyo 125-8585, Japan; 8115133@alumni.tus.ac.jp; 2 Autex Inc., 16-5 Tomihisa, Shinjuku, Tokyo 162-0067, Japan; s_hiwasa@autex-inc.co.jp

**Keywords:** ultraviolet nanoimprint lithography, durability, anisotropy, contact angle, line and space

## Abstract

Ultraviolet nanoimprint lithography (UV-NIL) requires high durability of the mold for the mass production of nanostructures. To evaluate the durability of a line-patterned replica mold made of high-hardness UV curable resin, repetitive transfer and contact angle measurements of the replica mold were carried out. In the line patterns, as the contact angle decreases due to repeated transfer, capillary action occurs, and water flows along them. Therefore, it can be said that a mold with a line pattern exhibits an anisotropic contact angle because these values vary depending on the direction of the contact angle measurement. Subsequently, these anisotropic characteristics were investigated. It was determined that it was possible to predict the lifetime of line-and-space molds over repeated transfers. As the transcription was repeated, the contact angle along the line patterns decreased significantly before becoming constant. Moreover, the contact angle across the line pattern decreased slowly while maintaining a high contact angle with respect to the contact angle along the line pattern. The contact angle then decreased linearly from approximately 90°. The mold was found to be macroscopically defect when the values of the contact angle along the line pattern and the contact angle across the line pattern were close. Predicting the mold’s lifetime could potentially lead to a shortened durability evaluation time and the avoidance of pattern defects.

## 1. Introduction

Ultraviolet nanoimprint lithography (UV-NIL) can be used to fabricate nanoscale patterns without many process steps [1]. It allows numerous nanoscale items to be manufactured at low cost and in high numbers [2,3]. The master mold is usually an expensive material, such as Si, which takes time and labor to process [4,5]. Therefore, it is necessary to prevent damage to the master mold due to resin adhesion. Coating the master mold with a release agent on the surface can prevent such damage [6].

However, repeated transfer degrades the mold release agent and eventually damages the master mold [6,7,8,9]. Methods for avoiding damage to the master mold include using a replica mold [10,11,12] and predicting the master mold’s lifetime. To the best of our knowledge, there are no studies on the prediction of mold lifetime; therefore, the durability of replica molds is often evaluated alternatively. Specifically, the durability can be evaluated by measuring the water contact angle, which changes with the water height because of the surface free energy of the mold [13,14]. In the line pattern, water flows along the lines as water height decreases; therefore, the line pattern exhibits contact angle anisotropy [15].

In the current study, the durability of line patterns with anisotropic contact angles was evaluated, and the anisotropy characteristics of the contact angle were investigated. A replica mold for evaluating durability was fabricated using a release-agent-free high-hardness resin.

**Figure 1:**
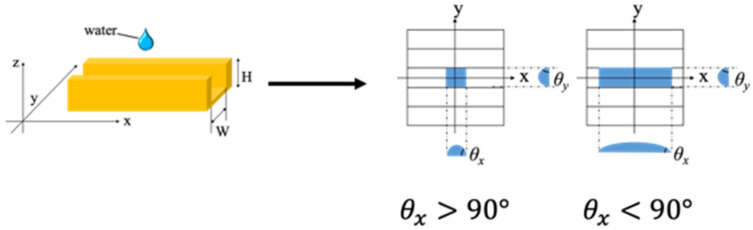
Contact angle measurements for the line-patterned replica mold.

As illustrated in Figure 1, the contact angle in the x direction ($\theta_{x}$), which is along the line pattern, and the contact angle in the y direction ($\theta_{y}$), which is across the line pattern, were measured. The anisotropy was then evaluated by comparing the measured $\theta_{x}$ and $\theta_{y}$.

## 2. Theory

Figure 1 depicts a schematic of the water flow on the line-patterned replica mold. The penetration distance of water in the capillary tube can be expressed as follows: The droplet position at the time of the contact angle measurement is considered as the origin position. The mold line direction, line width direction, and the height are represented by x, y, and z, respectively. First, the flow of water on a plane is expressed by an equation. The flow velocity is set to zero as the boundary condition between the fluid and the wall. The time term is omitted because the system is under steady flow conditions [16], and the external force term is omitted in the absence of any external force. When the fluid flows with a constant pressure gradient, the flow velocity of water is expressed by Equation (1) [17].

(1) $$u=\frac{1}{2\mu }\frac{\partial P}{\partial x}\left(y^{2}-Wy\right)=\frac{1}{2\mu }\frac{\Delta P}{l}\left(y^{2}-Wy\right)$$

where, ***u*** is the flow velocity, ***μ*** is the viscosity coefficient, ΔP is the pressure difference between the inside and outside of the liquid interface, ***W*** is the width of the second replica mold, ***l*** is the penetration distance, and ***H*** is the height. To obtain the water penetration distance, the flow rate, ***Q***, given in Equation (2) is obtained from Equation (1). The flow rate of the line-patterned structure is obtained by integrating the equation of the flow velocity by the width and multiplying the equation by the height. The resulting equation is as follows:

(2) $$Q=H\int_{0}^{W}u\;dy=-\frac{H}{12\mu }\frac{\partial P}{\partial x}W^{3}.$$

***Q*** can also be expressed by Equation (3).

(3) $$Q=\frac{dP}{dt}=\frac{WHdl}{dt}$$

Later, Equation (4) is obtained from Equations (2) and (3) and represents the water penetration distance:

$$-\frac{H}{12\mu }\frac{\Delta P}{l}W^{3}=\frac{WHdl}{dt}$$

$$ldl=-\frac{\Delta P}{12\mu }W^{2}dt$$

(4) $$l=\sqrt{\left\lceil \frac{\Delta P}{6\mu }W^{2}t\right\rceil }$$

where, *t* is the water penetration time and *l* is the water penetration distance. Furthermore, the pressure difference ΔP can be obtained from Equation (5) [17]. However, in the line-and-space (L&S) pattern, the top surface was open; consequently, the value of cosθ for the top surface was zero.

(5) $$\Delta P=\frac{\left(2H+W\right)\delta \cos\theta }{WH}$$

where, δ is the surface tension of water and θ is the contact angle. The pressure is positive or negative depending on the value of cosθ. Therefore, ΔP is negative when ***θ*** is greater than 90° and positive when θ is less than 90°. Moreover, the direction of the capillary force varies depending on whether θ is ≥90° or <90° [18].

## 3. Materials and Methods 

The first replica mold was fabricated [19] as follows: UV-curable resin, PAK-01-CL (Toyo Gosei Co., Ltd., Tokyo, Japan) was dropped on a Si master mold (Toppan Co., Ltd., Tokyo, Japan). PAK-01-CL is an acrylic base resin and a radical polymerization system. For this purpose, Si master molds with line widths of 100 nm and 200 nm were used. The periodicity of the first master mold was 100 nm line width and 100 nm spacing, whereas that of the second was 200 nm line width and 200 nm spacing. Subsequently, the PAK-01-CL was filled on the Si master mold by pressing the film (Cosmoshine A4300; Toyobo Co., Ltd., Osaka, Japan). The PAK-01-CL was then cured by UV irradiation at a dose of 120 mJ/cm^2^ using a UV lamp (Aicure UP50 (Panasonic Co., Ltd., Osaka, Japan), wavelength: 365 nm). The mold fabricated by release was labeled as the first replica mold.

**Figure 2:**
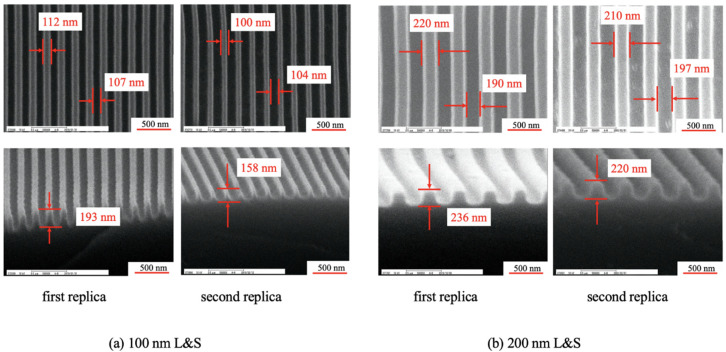
Scanning electron microscopy (SEM) images of the first and second replica molds for the transferred patterns of the (**a**) 100 nm line-and-space (L&S) and (**b**) 200 nm L&S transferred patterns.

After fabricating the first replica mold, mold release treatment was performed on its surface for improving the mold release. Specifically, 10-nm-thick Pt was deposited on the mold surface, and the mold was then immersed in Optool DSX 0.1% (Daikin Co., Ltd., Osaka, Japan) for 24 h. Later, heating was performed at 85 °C for 30 min to make the fluorinated hydrocarbon chains on the mold surface face upward [20]. The mold was then rinsed for 1 min with Optool HD-TH (Daikin Co., Ltd., Osaka, Japan). Finally, a second replica mold was fabricated from the first replica mold through UV-NIL. Specifically, a release-agent-free high-hardness resin (PARQIT OEX-066-X1-3; Autex Co., Ltd., Tokyo, Japan) was used for its fabrication [19]. This resin exhibits a viscosity in the range of 60–150 mPa·s at 23 °C, a pencil hardness of 9H, and a Young’s modulus of 1305 MPa. Because this resin was a release-agent-free resin, there was no need to perform release treatment on it [21]. A UV dose of 50 J/cm^2^ was provided by a UV lamp (Aicure UP50 (Panasonic Co.), wavelength: 365 nm). During UV irradiation, the mold was heated at 80 °C to promote curing [19]. After mold release, it was heated at 100 °C for 30 min to improve its mold release ability. These replica molds were prepared with 100 nm line-and-space (L&S) and 200 nm L&S patterns. The first and second replica molds were formed on a polyester film (COSMOSHINE 4300, Toyobo Co., Ltd., Osaka, Japan). The reason behind creating a replica mold of PAK-01-CL was to protect the silicon master mold. This was because PARQIT OEX-0066-X1-3 sometimes partially adhered to the silicon master mold surface even though the surface is released. In contrast, because PAK-01-CL exhibited decent release properties for the silicon master mold, we used the PAK-01-CL replica mold to fabricate the PARQIT OEX-0066-X1-3 replica mold. Figure 2 depicts the scanning electron microscopy (SEM) images of the first and second replica molds.

For the 100 nm L&S pattern, the first replica mold had a line width, space width, and a height of 112, 107, and 193 nm, respectively. The second replica mold had a line width, space width, and height of 100, 104, and 158 nm, respectively. Whereas in the 200 nm L&S pattern, the first replica mold had a line width, space width, and height of 220, 190, and 236 nm, respectively. The second replica mold had a line width, space width, and height of 210, 197, and 220 nm, respectively. The variation in height from the first replica mold to the second was caused by shrinkage, which occurred during curing [20].

**Figure 3:**
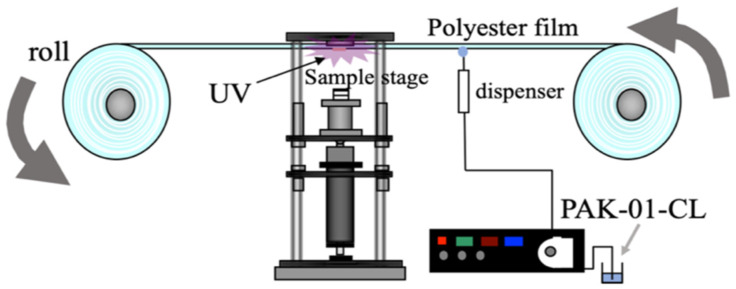
Transfer endurance and barge endurance device.

The durability of the second replica mold was then evaluated by repeating the transfer using the machine (Mitsui Electric Co., Ltd., Chiba, Japan) in Figure 3).

**Figure 4:**
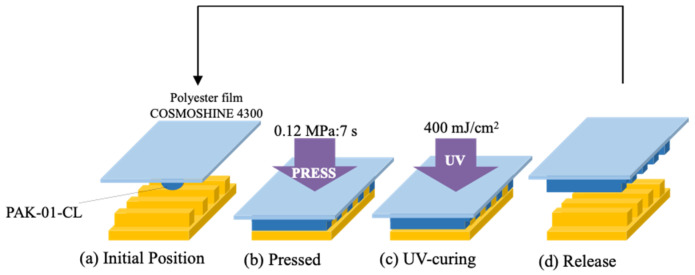
The process of repeated transcription.

Repeated transcription was performed as illustrated in Figure 4. PAK-01-CL was used as the resin during the durability test. After pressurizing at 0.12 MPa and filling the resin, UV irradiation with a dose of 400 mJ/cm^2^ was applied. In this case, a UV LED (ZUV -C20H (OMRON Co. Ltd., Kyoto, Japan), wavelength: 365 nm) was used for UV curing. The study was also conducted at a temperature of 21 °C.

To measure the release ability of the second replica mold, the water contact angle was measured (Drop master-701, Kyowa Interface Science Co., Ltd., Niiza City, Japan) at various imprint numbers. The water droplet volume at the time of contact angle measurement was 2 μL.

**Figure 5:**
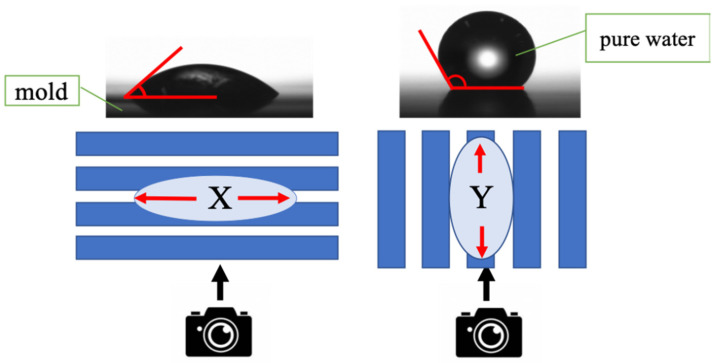
Measurement of the contact angle between x and *y*.

The contact angle was measured every 20 times from the 1st to 100th cycles, every 50 times from the 100th to 400th cycles, every 100 times from the 400th to 1000th cycles, every 200 times from the 1000th to 3000th cycles, and every 250 times thereafter. Moreover, the contact angle was measured five times at the center of the mold after a predetermined number of transfers. It was confirmed that the standard deviation of these five data was less than 3.0°. The structure being evaluated for durability was line-patterned; therefore, a capillary force acted while measuring the contact angle. Water was deposited and the contact angles in the x and y directions were measured after 3 s, as shown in Figure 1. Furthermore, because water flowed in the direction of the fine lines due to the capillary force, the contact angle varied depending on the measurement direction. In order to evaluate the anisotropy, contact angle measurements were made from two directions, as illustrated in Figure 5.

## 4. Results and Discussion

**Figure 6:**
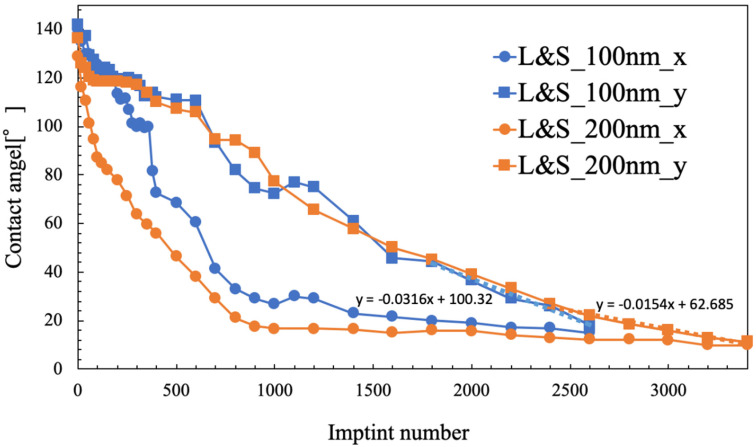
Contact angles after different numbers of imprints.

Figure 6 shows the results of the durability evaluation of the second replica mold, specifically the contact angles for the 100 nm and 200 nm L&S patterns. There were some similarities between the results obtained for these L&S widths. The contact angle was approximately 140° before the transfers, which eventually decreased with repeated transfers. Moreover, as the contact angle decreased, water began to flow along the line pattern; specifically, the contact angle in the x direction decreased to approximately 20° before it stabilized. On the other hand, the contact angle in the y direction decreased in a more linear manner. In the 100 nm L&S pattern, the contact angle in the y direction was linear after 1800 transfers with a gradient of −0.0316. In the 200 nm L&S pattern, the contact angle in the y direction was linear after 2400 transfers with a gradient of −0.0154. This gradient halved when the scale between the lines of the L&S pattern was doubled. A linear contact angle in the y direction corresponded to a smaller L&S scale, and the defect was likely to form relatively sooner.

The experimental results indicated that the contact angle in the x direction became constant at approximately 20°. The point where the above-mentioned inclination and approximately 20° straight line intersected was predicted to be at 2600 imprints for the 100 nm L&S pattern and at 3400 imprints for the 200 nm L&S pattern. For the 100 nm L&S pattern, a defect occurred in the second replica mold at approximately transfer number 2600. For the 200 nm L&S pattern, a defect occurred in the second replica mold at approximately transfer number 3400. We categorized the lifetime of the replica mold before observing the macroscopic defect. In the line pattern mold, capillary force is generated along the line pattern, and this force spreads the water along the line direction (x direction). As a result, the contact angle observed in the x direction was lower than that measured across the line direction (y direction). Here, we assumed the mold surface energy to be constant and that the contact angle of a regular pattern (such as dots or holes) and the contact angles of x and y directions are equal. In contrast, in the case of line patterns, the direction of capillary force enhances the water spreading and exhibits lower contact angles. Furthermore, we think that the change in contact angles caused by the capillary flow indicates the future contact angle in the other direction (in this case the y direction). As shown in Figure 6, the contact angle in the x direction was saturated at 20°. This means that the contact angle in the y direction can decrease to this value (20°) without forming any macroscopic defects, because the x direction exhibited no macroscopic defects at the contact angle of 20°.

**Figure 7:**
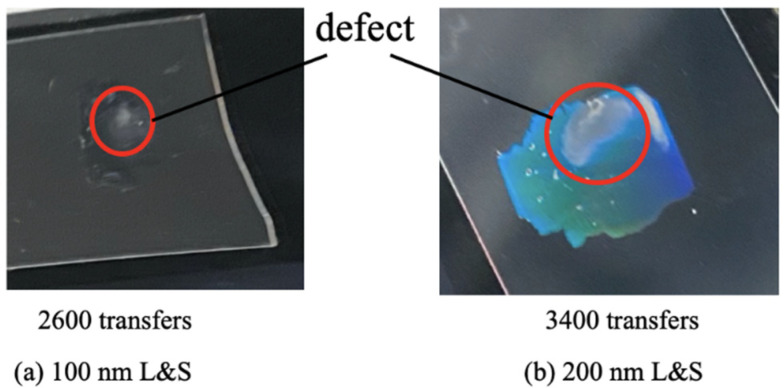
Images of a defect of the (**a**) 100 nm L&S and (**b**) 200 nm L&S transferred patterns.

**Figure 8:**
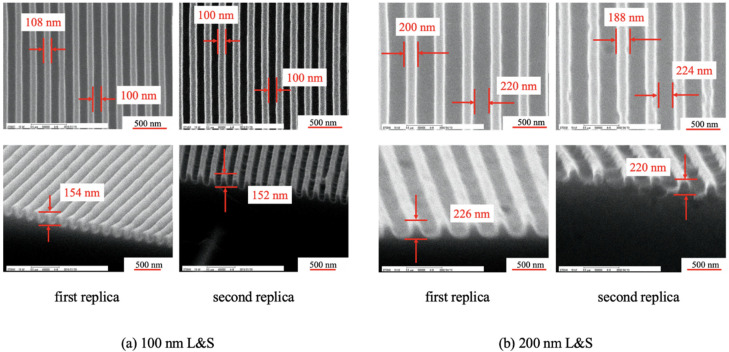
SEM images of the (**a**) 100 nm L&S and (**b**) 200 nm L&S transferred patterns.

We observed the macroscopic defect as shown in Figure 7. Figure 8 shows SEM images of the transferred film surface using PAK-01-CL.

In the 100 nm L&S pattern, the first transfer had a line width, space width, and a height of 108, 102, and 154 nm, respectively. The corresponding values observed for transfer number 2400 were 100, 100, 152 nm, respectively. In the 200 nm L&S pattern, the first transfer had a line width, space width, and height of 200, 220, and 226 nm, respectively. The corresponding values observed for transfer number 3200 were 188, 224, and 220 nm, respectively.

**Figure 9:**
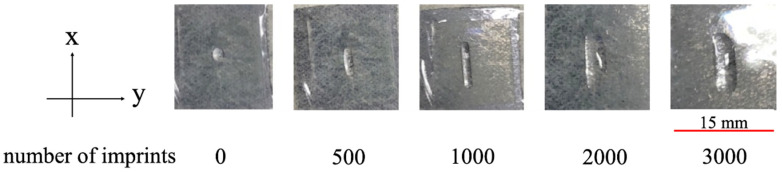
Water penetration distance for various imprint numbers.

Figure 9 depicts how the contact angle decreased linearly in the y direction and approached a constant value in the x direction. The second replica mold included fluorinated materials. We considered that these release materials are gradually removed during repetition of the UV-NIL process. This is the reason for the linear decrease in the y direction (see Figure 6). Moreover, the contact angle in the x direction becomes constant at an earlier stage as compared to that in the y direction. Therefore, the intersection of x and y can be predicted. Furthermore, a visible deficit in the number of transcriptions near the intersection of x and y was observed. From these results, it is possible to predict the approximate number of transcriptions after which the defect occurs by predicting the intersection of x and y. The lifetime of the line-patterned mold could be predicted because the contact angle differed in the x and y directions. It is thought that this lifetime was the result of water that flows along the line pattern due to capillary force.

**Figure 10:**
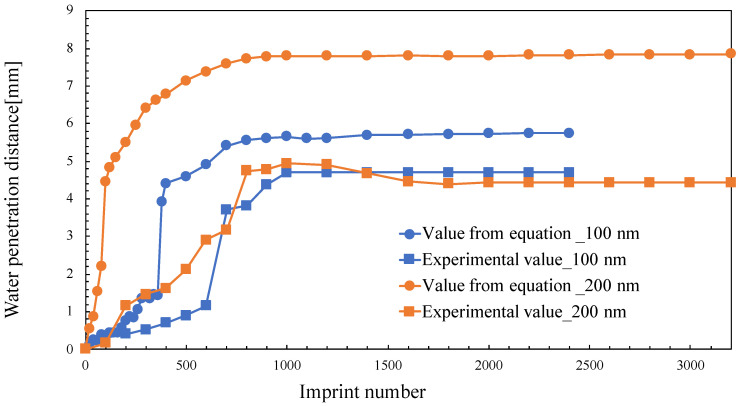
Water penetration distance with increasing imprint number.

Figure 10 shows the water penetration distance for various numbers of imprints. The penetration distance in the y direction increased with increasing transfer number. The penetration distance was calculated while considering the direction of the force. The values of ***W*** and ***H*** were obtained from Figure 2. ***μ*** is the viscosity of PAK-01-CL (1.002 mPa·s) and ***δ*** is the surface tension of water (7.225 × 10^−5^ N/mm), which is used for calculating **Δ*P***, as expressed in Equation (5). Considering the sign of cosθ, the values provided in Table 1 were substituted into Equation (5). The penetration distance was obtained by substituting the obtained pressure ΔP into Equation (4).

The measured experimental values were compared with those calculated using Equation (4). As shown in this figure, the values of water penetration distances obtained from the experiment and those evaluated from the equations were different. However, leveling off was generated after approximately 1000 imprints, which means that saturation of contact angle occurred after approximately 1000 repetitions. Therefore, the minimum requirement of lifetime prediction is approximately 1000 repetitions. Furthermore, the penetration distance between the lines was calculated. However, in practice, water on the lines exhibits the Cassie–Baxter and Wenzel states [22]. The calculated values assumed that all the water was in between the lines. In reality, the difference was caused by the presence of water above these lines as well. When the transfer was repeated, the penetration distance became constant. This was because the pressure depended on the contact angle. Moreover the theoretical and experimental values displayed similar plots; therefore, it can be said that capillary action occurs within the molds in this experiment. It can hence be concluded that the contact angle anisotropy of the molds with the line pattern exists due to this capillary action.

## 5. Conclusions

Ultraviolet nanoimprint lithography (UV-NIL) can be used to fabricate nanoscale patterns while ensuring a high throughput and low cost. The high-volume production of nanostructures requires the durability of the release-coated silicon mold to be excellent. Accordingly, the durability of a line-patterned mold was evaluated using a high-hardness release-agent-free resin. Capillary action occurred in the nanoscale line structure. It was observed that the contact angle decreased with repeated transfers and that the water flowed along the lines. Measuring the contact angle in the x and y directions allowed the prediction of the lifetime of the mold. This prediction method is limited to line-and-space patterns. However, using line-and-space patterns, every release coating or release material can be evaluated. In addition, the minimum number of UV-NIL imprints required to estimate the stamp lifetime is until saturation of the contact angle in the x direction occurs. We obtained the contact angles in both x and y directions vs. the number of imprints. Lifetime can be estimated from the crossing point of the x direction (saturated low contact angle value) curve and the corresponding y direction curve. This is a facile and labor-saving way. This lifetime prediction could potentially lead to a shortening of the durability evaluation time and prevention of mold breakage. If this comparison of x and y in L&S patterns, or life prediction, can be utilized, it will be possible to determine which demolding process is best for a smaller number of transfers.

## Figures and Tables

**Table 1:** Values assigned to Equation (5) for water penetration distance.

Scale [nm]	*W* [nm]	*H* [nm]	*t* [s]
100	104	158	5
200	197	220	5
